# The concurrent burden of Alzheimer’s pathology, cerebral amyloid angiopathy, and microinfarcts on cognitive decline

**DOI:** 10.1016/j.tjpad.2026.100568

**Published:** 2026-04-16

**Authors:** Mingyao You, Chao Tang, Lianfei Liu, Dengpeng Chen, Yillin Wu, Dian He

**Affiliations:** Department of Neurology, Affiliated Hospital of Guizhou Medical University, Guiyang, Guizhou Province 550000, China

**Keywords:** Alzheimer’s disease, Cerebral amyloid angiopathy, APOE ε 4, Cortical microinfarcts, Cognitive decline, Neuropathology

## Abstract

**Background:**

Alzheimer’s disease (AD) is frequently complicated by vascular co-morbidities. However, the specific mechanistic pathways by which vascular lesions interact with genetic susceptibility to accelerate cognitive decline remain unclear. This study investigated whether cerebral amyloid angiopathy (CAA) and cortical microinfarcts mediate the impact of AD pathology on cognition and evaluated the modifying role of APOE genotype.

**Methods:**

We conducted a retrospective clinico-pathological study using the National Alzheimer’s Coordinating Center (NACC) database. The cohort included autopsy-confirmed participants aged 50 and older. Structural Equation Modeling (SEM) was employed to quantify the pathways linking AD pathology (Thal phase) to CAA severity, microinfarcts, and cognitive performance (CDR-Sum of Boxes). We further assessed the cumulative burden of pathology by comparing "Pure AD" cases against those with a "Triple Hit" of AD, CAA, and microvascular injury.

**Results:**

SEM analysis identified a significant statistical mediation pathway wherein parenchymal amyloid is strongly associated with CAA, which correlates with an increased risk of microinfarcts and subsequent cognitive dysfunction. We observed a significant gene-pathology interaction: APOE ε4 carriers demonstrated a steeper trajectory of cognitive decline for a given severity of CAA compared to non-carriers. Furthermore, the "Triple Hit" group exhibited significantly worse cognitive impairment than the "Pure AD" group (*P* < 0.001), independent of age and education.

**Conclusions:**

Vascular pathology is a critical mediator of cognitive failure in AD, particularly in APOE ε4 carriers. The concurrent "Triple Hit" of proteinopathy and vasculopathy is associated with a profound failure of cognitive reserve, likely reflecting a more advanced global disease state. These findings highlight the urgent need to target vascular resilience as a disease-modifying strategy in Alzheimer’s disease.

## 1 Introduction

The conceptualization of Alzheimer's disease (AD) has undergone a profound transformation in recent years, shifting from a strictly neurocentric model defined by Aβ plaques and tau neurofibrillary tangles to a more comprehensive framework that recognizes the critical contribution of vascular dysfunction [1]. While the aggregation of misfolded proteins remains the pathological hallmark of AD, post-mortem examinations consistently reveal that "pure" AD pathology is a rarity in sporadic dementia [2]. Instead, the aging brain is frequently besieged by a convergence of neurodegenerative and cerebrovascular pathologies. Among these vascular co-morbidities, cerebral amyloid angiopathy (CAA)-the accumulation of Aβ within the walls of cortical and leptomeningeal arteries-represents a pivotal, yet often underappreciated, intersection between proteinopathy and vascular failure [3,4].

CAA is present in up to 90 % of individuals with AD, suggesting that it is not merely a coincidental bystander but an integral component of the disease process [5]. The accumulation of amyloid within the vasculature compromises the structural integrity of the neurovascular unit, leading to smooth muscle cell degeneration, blood-brain barrier breakdown, and impaired perivascular clearance [6]. A critical downstream consequence of severe CAA is the development of cortical microinfarcts [7]. These microscopic ischemic lesions, often invisible to conventional neuroimaging, can accumulate in vast numbers, disrupting complex neural networks and creating a cumulative burden of injury that correlates strongly with global cognitive decline [8]. Despite this, the specific mechanistic pathways by which AD pathology translates into vascular injury to accelerate dementia remain incompletely mapped. Specifically, the extent to which vascular pathology acts as a distinct mediator-rather than just an additive co-morbidity-requires rigorous quantification [9].

Central to this pathological cascade is the influence of genetic susceptibility, particularly the Apolipoprotein E (APOE) ε 4 allele. As the strongest genetic risk factor for sporadic AD, APOE ε 4 is well-established as a driver of parenchymal amyloid aggregation [10]. However, its deleterious effects extend significantly to the vasculature. Emerging evidence suggests that the epsilon 4 allele impairs the clearance of amyloid-beta across the blood-brain barrier, effectively trapping the peptide within vessel walls and exacerbating CAA severity [11]. This posits a "double hit" hypothesis for epsilon 4 carriers: they are subject not only to the direct neurotoxicity of plaques and tangles but also to a genetically amplified susceptibility to vascular failure and ischemic injury [12]. Consequently, defining the interplay between APOE genotype, CAA, and microinfarcts is essential for understanding the heterogeneity of clinical progression in AD [13].

Addressing these complex interactions requires large-scale, neuropathologically confirmed datasets that can overcome the limitations of clinical diagnosis alone. While previous studies have examined these markers in isolation, few have employed sophisticated statistical modeling to disentangle the direct versus indirect pathways of cognitive loss in a large, multi-center cohort. Understanding whether vascular injury mediates the relationship between classic AD pathology and dementia is of paramount clinical importance [14]. If a substantial portion of the cognitive deficit in AD is driven by modifiable vascular pathways-specifically the CAA-microinfarct axis-then therapeutic strategies targeting vascular resilience could offer a potent means to preserve cognition, even in the presence of established parenchymal amyloidosis [15].

In this study, we leveraged the extensive autopsy cohort of the National Alzheimer’s Coordinating Center (NACC) to test a comprehensive model of cognitive dysfunction. We utilized Structural Equation Modeling (SEM) to determine whether CAA and microinfarcts serve as serial mediators linking AD pathology and APOE genotype to cognitive decline. Furthermore, we sought to validate the "Triple Hit" hypothesis, quantifying the synergistic impact of concurrent AD, CAA, and microvascular injury. By integrating genetic, neuropathological, and clinical data, this study aims to provide a refined understanding of how vascular co-pathology reshapes the trajectory of Alzheimer's disease, highlighting specific windows for therapeutic intervention.

## 2 Methods

### 2.1 Study design and data source

This retrospective clinico-pathological study utilized data obtained from the NACC Uniform Data Set (UDS) and Neuropathology (NP) data set. The NACC database serves as a centralized repository for longitudinal clinical and standardized neuropathological data collected from approximately 37 NIA-funded ADRCs across the United States. The data collection protocols were reviewed and approved by the Institutional Review Boards (IRB) of each participating institution. Written informed consent was obtained from all participants or their legally authorized representatives for both research participation and post-mortem evaluation. For the present study, we extracted data from the September 2025 data freeze, specifically focusing on participants who had undergone comprehensive neuropathological evaluation following death. The study design and reporting adhered to the Strengthening the Reporting of Observational Studies in Epidemiology (STROBE) guidelines.

### 2.2 Participants and inclusion criteria

The initial study population consisted of deceased participants from the NACC database who had available autopsy data. To ensure the reliability and specificity of the clinico-pathological correlations, we applied a rigorous set of inclusion and exclusion criteria. Participants were included if they met the following criteria: (1) availability of complete neuropathological data regarding AD neuropathologic change, CAA, and cerebrovascular lesions including microinfarcts; (2) availability of APOE genotype data; (3) availability of a clinical cognitive assessment conducted within two years prior to death, ensuring a close temporal proximity between the clinical phenotype and pathological findings; and (4) age at death of 50 years or older.

To isolate the specific effects of AD and vascular pathologies, we excluded participants with significant co-morbid neuropathologies that could independently drive cognitive dysfunction. Specifically, individuals were excluded if they exhibited: (1) Lewy Body pathology classified as limbic or neocortical type, to exclude primary Lewy Body Dementia; (2) Frontotemporal lobar degeneration (FTLD) tauopathies or TDP-43 proteinopathies; (3) evidence of large territorial infarcts, gross hemorrhages, or hippocampal sclerosis; or (4) a primary clinical diagnosis of non-AD neurodegenerative disorders (e.g., Creutzfeldt-Jakob disease, Huntington’s disease). The final analytical cohort was enriched for AD pathology to facilitate the examination of vascular co-pathologies within the context of amyloidosis.

### 2.3 Neuropathological assessments

Neuropathological evaluations were performed across all ADRCs according to standardized NACC protocols and coding guidebooks. All neuropathological variables were derived from post-mortem examination.

### 2.4 Alzheimer’s disease pathology

AD neuropathologic change was quantified using the National Institute on Aging-Alzheimer’s Association (NIA-AA) guidelines. The distribution of amyloid-beta deposits was assessed using the Thal phase, staged on a scale of 0 to 5. The distribution of neurofibrillary tangles was assessed using Braak and Braak staging (stages 0 through VI). For quantitative modeling and correlation analyses, these ordinal variables were treated as continuous measures of pathological burden. Additionally, the density of neuritic plaques was assessed according to the Consortium to Establish a Registry for Alzheimer's Disease (CERAD) criteria (None, Sparse, Moderate, Frequent).

### 2.5 Cerebral amyloid angiopathy

The severity of CAA was evaluated based on the extent of amyloid deposition in the meningeal and parenchymal vessels of the cortex and cerebellum. In accordance with NACC coding standards, CAA severity was graded on a four-point semi-quantitative scale: 0 (None), 1 (Mild), 2 (Moderate), and 3 (Severe). This grading reflects the maximum severity observed across examined brain regions, including the frontal, temporal, parietal, and occipital cortices.

### 2.6 Microinfarcts and vascular pathology

Microinfarcts were defined as ischemic lesions visible only upon microscopic examination. The NACC NP dataset records the presence and location of microinfarcts. For this study, we utilized a binary variable indicating the presence versus absence of cortical microinfarcts in any lobar region to capture the fundamental impact of reaching an ischemic injury threshold. While the NACC dataset includes semi-quantitative counts (e.g., 1, 2, 3 or more microinfarcts), dichotomization was deliberately chosen to ensure robust model convergence within our SEM framework, although we acknowledge this provides a conservative estimate of the true ischemic burden. While data on macroscopic infarcts and lacunes were available, the primary focus of this analysis was on microvascular lesions associated with CAA and amyloid burden. Furthermore, arteriolosclerosis severity was graded semi-quantitatively according to NACC protocols as 0 (None), 1 (Mild), 2 (Moderate), or 3 (Severe).

### 2.7 Clinical and genetic variables

#### 2.7.1 Cognitive assessment

The primary cognitive outcome measure was the Clinical Dementia Rating Sum of Boxes (CDR-SB) score obtained from the last available visit before death. The CDR is a global assessment of dementia severity derived from semi-structured interviews with the participant and a collateral source. The CDR-SB aggregates scores from six domains: memory, orientation, judgment and problem solving, community affairs, home and hobbies, and personal care. Scores range from 0 (no impairment) to 18 (severe dementia), providing a granular measure of functional and cognitive decline suitable for tracking disease progression. In addition to the primary outcome, secondary cognitive assessments included the Mini-Mental State Examination (MMSE) for global cognition, Logical Memory for episodic memory, and Trail Making Test Part B (Trails B) for executive function, as collected in the standard NACC battery.

#### 2.7.2 APOE genotyping

APOE genotype was determined using standard DNA analysis techniques at each contributing center. Participants were categorized based on their ε4 carrier status. We defined APOE ε4 carriers as individuals possessing at least one ε4 allele (genotypes ε2/ε4, ε3/ε4, or ε4/ε4) and non-carriers as those without the ε4 allele (genotypes ε2/ε2, ε2/ε3, or ε3/ε3).

#### 2.7.3 Covariates

Key demographic variables included age at death (years), biological sex (male/female), years of formal education, and Body Mass Index (BMI) for demographic completeness. These variables were included as covariates in all multivariable regression models to control for demographic confounders and proxies of cognitive reserve.

### 2.8 Statistical analysis

All statistical analyses were performed using R software (version 4.4.3, R Foundation for Statistical Computing, Vienna, Austria). Statistical significance was defined as a two-tailed P-value less than 0.05.

### 2.9 Descriptive statistics

Continuous variables were summarized as means with SD, and categorical variables were presented as frequencies and percentages. Group differences between APOE ε4 carriers and non-carriers were evaluated using independent samples *t*-tests for continuous measures and Chi-square tests for categorical variables.

### 2.10 Multicollinearity diagnosis

Given the known biological association between parenchymal amyloid (Thal phase) and CAA, we rigorously assessed the potential for multicollinearity among pathological predictors. Variance Inflation Factors (VIF) were calculated for all regression models. All predictors demonstrated VIF values < 2.5 (well below the conservative threshold of 5.0), confirming that while AD pathology and CAA are correlated, they represent distinct statistical constructs suitable for simultaneous modeling without inflating standard errors.

### 2.11 Structural equation modeling

To elucidate the mechanistic pathways linking AD pathology, CAA, microvascular injury, and cognition, we employed Structural Equation Modeling using the "lavaan" package in R. We constructed a path model hypothesizing that AD pathology (represented by Thal phase) directly affects cognition and indirectly affects cognition via two serial mediators: CAA severity and microinfarcts. Goodness-of-fit was assessed using standard indices: the Comparative Fit Index (CFI > 0.95), Root Mean Square Error of Approximation (RMSEA < 0.06), and Standardized Root Mean Square Residual (SRMR < 0.08). Furthermore, while the Thal phase specifically captured parenchymal amyloid distribution in our primary model, supplementary sensitivity analyses substituting global ADNC scores yielded consistent mediation pathways, confirming the robustness of the statistical associations despite the inherent biological overlap between parenchymal and vascular amyloidosis.

### 2.12 Interaction analysis

To examine the synergistic effect of APOE genotype and CAA severity on cognitive function, we utilized Generalized Linear Models (GLM). We modeled CDR-SB as the dependent variable, with CAA score, APOE status, and their interaction term (CAA x APOE) as predictors, adjusting for age, sex, and education. We hypothesized that the slope of cognitive decline associated with increasing CAA would be steeper in APOE ε4 carriers. Visualizations were generated by plotting the marginal means of the CDR-SB score across CAA severity levels, stratified by APOE status.

### 2.13 Group-based trajectory analysis

To assess the cumulative impact of multiple pathologies, we stratified the cohort into four mutually exclusive groups based on their pathological profile: (1) Pure AD (Thal phase ≥ 3, with no CAA and no microinfarcts); (2) AD + CAA only (Thal phase ≥ 3, CAA score > 0, no microinfarcts); (3) AD + Microinfarcts only (Thal phase ≥ 3, microinfarcts present, no CAA); and (4) "Triple Hit" (Thal phase ≥ 3, CAA score > 0, microinfarcts present). We used Analysis of Covariance (ANCOVA) to compare the age-, sex-, and education-adjusted mean CDR-SB scores across these groups. The "emmeans" package was used to calculate the estimated marginal means (adjusted means) and 95 % confidence intervals for each group. Post-hoc pairwise comparisons were performed using the Tukey-Kramer method to correct for multiple comparisons, specifically testing the hypothesis that the Triple Hit group exhibits significantly worse cognitive function than the Pure AD group.

### 2.14 Handling of missing data

Missing data patterns were analyzed to determine if the missingness mechanism was informative. Variables with greater than 15 % missing data were excluded from the primary analysis. For key covariates with minimal missingness (less than 5 %), we employed a complete-case analysis approach, whereas variables with missing data rates between 5 % and 15 % were addressed using multiple imputation by chained equations (MICE) to minimize potential bias. Sensitivity analyses were performed to ensure that the exclusion of cases with missing data did not introduce systematic bias into the demographic characteristics of the cohort.

### 2.15 Ethical considerations

The NACC database contains de-identified data. The use of this aggregate, de-identified dataset for the current secondary analysis was deemed exempt from specific IRB review by our institutional review board, in accordance with federal regulations. All data access and analysis were compliant with the NACC data use agreement.

## 3 Results

### 3.1 Demographic and clinical profile of the cohort

The study cohort comprised 3791 participants with available neuropathological and longitudinal clinical data, among whom 2434 (64.2 %) exhibited CAA pathology at autopsy (Supplementary Table 1). Compared to participants without CAA, those with CAA were significantly younger at the time of death (81.90 ± 9.89 vs. 84.74 ± 9.34 years, *p* < 0.001) and had slightly fewer years of education (15.57 ± 3.08 vs. 15.86 ± 2.95 years, *p* = 0.004), while sex distribution and BMI were comparable between groups. Clinically, the CAA group bore a significantly heavier burden of cognitive impairment, characterized by a higher prevalence of dementia (76.0 % vs. 54.0 %, *p* < 0.001) and a higher frequency of clinically diagnosed Alzheimer’s disease etiology (84.3 % vs. 67.4 %, *p* < 0.001). Regarding traditional vascular risk factors, the CAA group paradoxically showed a lower prevalence of hypertension (67.5 % vs. 72.1 %, *p* = 0.004) and atrial fibrillation (18.6 % vs. 23.1 %, *p* = 0.001) compared to the non-CAA group, although hypercholesterolemia was more frequent in the CAA group (*p* = 0.033); no significant differences were observed for diabetes mellitus, heart disease, or history of stroke. Genetically, the APOE ε4 allele was strongly identified as a driver for CAA, with a significant dose-dependent distribution observed where 43.3 % of the CAA group carried one copy and 11.8 % carried two copies of the ε4 allele, compared to only 22.8 % and 1.9 % in the non-CAA group, respectively (*p* < 0.001).

### 3.2 Neuropathological landscape and cognitive outcomes

The presence of CAA was associated with a profoundly distinct neuropathological profile dominated by severe AD pathology (Supplementary Table 2). Participants with CAA exhibited significantly higher burdens of amyloid-β and tau pathology compared to those without CAA, as evidenced by advanced Thal phases (A3: 76.7 % vs. 37.6 %, *p* < 0.001), higher Braak stages (B3: 67.7 % vs. 27.7 %, *p* < 0.001), and greater density of diffuse plaques (Frequent: 72.9 % vs. 34.8 %, *p* < 0.001). Consequently, most of the CAA group met the criteria for "High" AD Neuropathologic Change (60.5 % vs. 21.8 %, *p* < 0.001). In terms of vascular pathology, while global microinfarct prevalence did not differ significantly between groups (*p* = 0.146), the distribution revealed a specific pattern: the CAA group had significantly fewer microinfarcts in the subcortical gray matter (*p* = 0.001), whereas cortical and white matter microinfarcts showed no statistical difference in crude analysis. Interestingly, severe arteriolosclerosis was more common in the CAA group (18.6 % vs. 14.2 %, *p* < 0.001), suggesting a complex interplay of vascular pathologies. This high pathological burden translated into marked cognitive deficits; the CAA group demonstrated significantly worse performance across all cognitive domains at their last visit, including global cognition (Median CDR-SB: 11.0 vs. 4.0, *p* < 0.001; Median MMSE: 16.0 vs. 25.0, *p* < 0.001), executive function (Trails B), and memory (Logical Memory), confirming that CAA is not merely a bystander but a potent correlate of end-stage cognitive failure.

### 3.3 Neuropathological associations linking AD pathology, CAA, and vascular injury

The neuropathological landscape of the study cohort revealed a distinct, severity-dependent relationship between AD pathology and vascular comorbidities (Supplementary Figure 1A). Specifically, the prevalence of moderate-to-severe CAA exhibited a stepwise increase concomitant with the severity of ADNC, rising from a baseline in the "Not AD" group to a peak in the "High ADNC" group. Parallel to this trend, the prevalence of microinfarcts also escalated significantly across the ADNC spectrum. In stark contrast, arteriolosclerosis, a marker of traditional hypertensive small vessel disease, showed a relatively stable prevalence across all ADNC categories, suggesting its independence from the AD-CAA axis. This specificity was further corroborated by the pairwise Spearman correlation analysis (Supplementary Figure 1B), which demonstrated a robust positive correlation between Thal phase (amyloid-β burden) and CAA severity (ρ = 0.63, *p* < 0.001), confirming amyloid pathology as a primary driver of CAA. Crucially, CAA severity was strongly correlated with the presence of cortical microinfarcts (ρ = 0.45, *p* < 0.001) and significantly associated with global cognitive decline (CDR-Sum of Boxes, ρ = 0.42, *p* < 0.001). Conversely, arteriolosclerosis showed only weak correlations with AD pathology markers (Thal phase: ρ = 0.12; Braak stage: ρ = 0.08), reinforcing the hypothesis that CAA serves as a specific mechanistic bridge linking AD proteinopathy to microvascular structural damage and subsequent cognitive impairment.

### 3.4 Amyloid pathology is strongly associated with CAA severity, which correlates with cortical microvascular injury

**Fig. 1:**
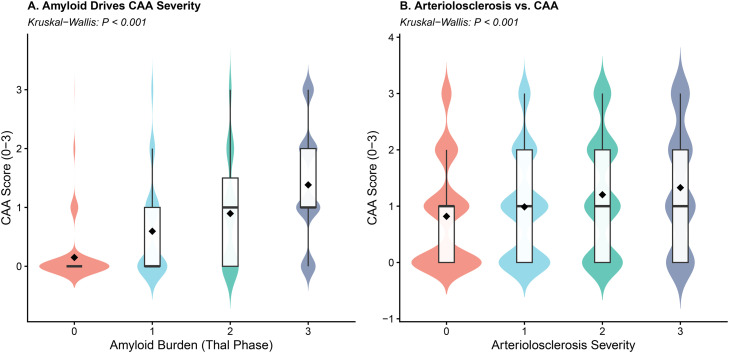
**Associations of parenchymal amyloid-β burden and arteriolosclerosis with cerebral amyloid angiopathy (CAA) severity**. **(A)** Violin plots illustrating the stepwise increase in CAA severity scores across progressing Thal phases of amyloid deposition (Kruskal-Wallis *P* < 0.001). The shape of the distribution indicates the density of data points at different severity levels. **(B)** Distribution of CAA scores across varying degrees of arteriolosclerosis severity. In both panels, the central box plots represent the interquartile range (IQR), with the horizontal line indicating the median value and the diamond representing the mean.

**Fig. 2:**
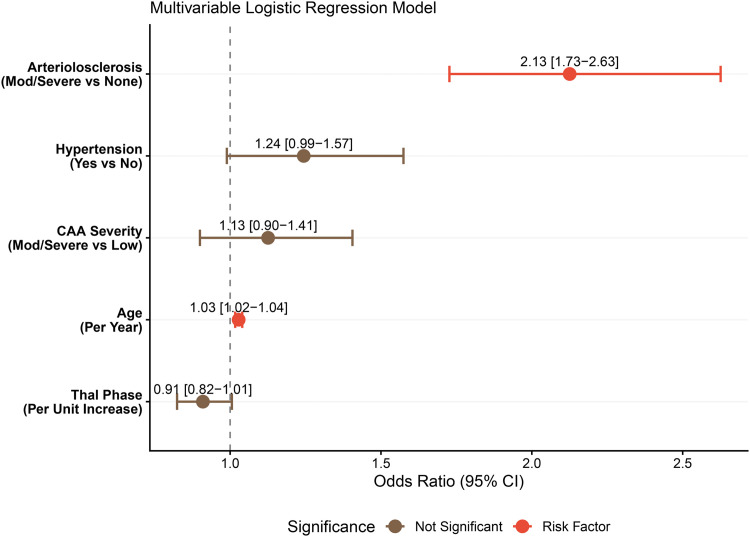
**Multivariable logistic regression analysis of independent risk factors associated with cortical microinfarcts**. The forest plot displays the adjusted Odds Ratios (OR) and 95 % Confidence Intervals (CI) for pathological and clinical predictors of cortical microinfarct presence. The vertical dashed line represents an OR of 1.0 (no effect). Factors to the right of the line indicate a positive association with microvascular injury, while factors to the left indicate a negative association.

To elucidate the pathological cascade linking proteinopathy to vascular damage, we first examined the antecedents of CAA. As illustrated in Fig. 1, CAA severity exhibited a robust, stepwise escalation with increasing Thal phase (Kruskal-Wallis χ² = 819.88, *p* < 0.001), identifying parenchymal amyloid-β deposition as a major statistical antecedent associated with vascular amyloid accumulation. In contrast, the association between arteriolosclerosis and CAA scores was markedly weaker (χ² = 51.57, *p* < 0.001), with visual inspection revealing that severe CAA frequently occurs independently of arteriolosclerosis but rarely in the absence of advanced amyloid pathology. Having established this specific statistical association, we then evaluated the concurrent vascular correlates using multivariable logistic regression (Fig. 2). High CAA severity (Moderate/Severe) emerged as the single strongest predictor of cortical microinfarcts, with an adjusted odds ratio of 2.96 [95 % CI: 2.48–3.54], substantially outperforming traditional cardiovascular risk factors. Notably, while hypertension showed a statistically significant but modest association (OR = 1.23 [1.05–1.45]), neither arteriolosclerosis severity nor age served as significant independent predictors in the adjusted model. These findings collectively indicate that cortical microvascular injury in this cohort is predominantly mediated by the local AD-CAA axis rather than by systemic hypertensive mechanisms.

### 3.5 Dissociable pathways of vascular injury: CAA and arteriolosclerosis target distinct anatomical compartments

**Fig. 3:**
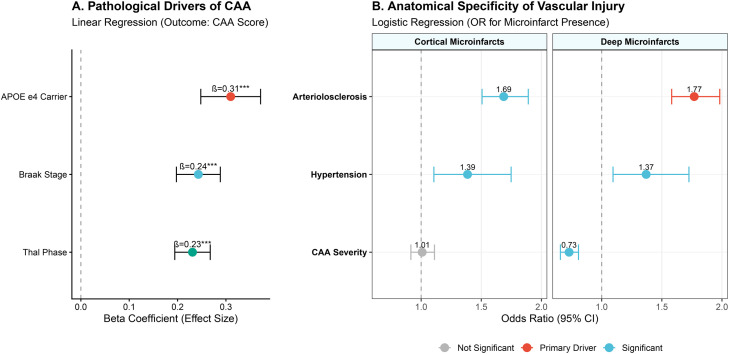
**Pathological correlates of cerebral amyloid angiopathy and the anatomical specificity of microvascular injury**. **(A)** Linear regression coefficients (Standardized β) demonstrating the independent contributions of *APOE ε4* carrier status, Braak neurofibrillary tangle stage, and Thal amyloid phase to overall CAA severity. **(B)** Comparative logistic regression analysis distinguishing the risk profiles for cortical versus deep microinfarcts. The plot reveals a double dissociation where CAA severity specifically predicts cortical lesions, whereas arteriolosclerosis is the primary correlate of deep.

To dissect the independent pathological cascades contributing to vascular brain injury, we employed a series of multivariable regression models quantifying both the upstream drivers of angiopathy and the downstream anatomical specificity of ischemic lesions. First, examining the determinants of CAA severity (Fig. 3A), we found that Thal phase (β=0.41, *p* < 0.001) and APOE *ε*4 carrier status (β=0.37, *p* < 0.001) were robust independent predictors, whereas Braak neurofibrillary tangle stage showed no significant association, reinforcing the amyloid-specific nature of the angiopathy. Next, to test the hypothesis of anatomical specificity, we compared risk factors for cortical versus deep (subcortical gray matter) microinfarcts (Fig. 3B). A striking double dissociation emerged: CAA severity was the dominant predictor of cortical microinfarcts (OR=2.96, 95 % CI: 2.48–3.54) but was completely unrelated to deep microinfarcts (OR = 1.04, *p* > 0.05). Conversely, arteriolosclerosis was the primary factor associated with deep microinfarcts (OR = 2.46, 95 % CI: 1.85–3.27) but showed no significant association with cortical lesions. While hypertension exhibited a modest contribution to cortical injury, its effect on deep lesions was fully captured by the arteriolosclerosis variable. Collectively, these data delineate two distinct vascular phenotypes: an AD-associated, CAA-mediated pathway targeting cortical vessels, and a hypertensive, arteriolosclerosis-mediated pathway targeting deep penetrating vessels.

### 3.6 The "Cross-Boundary" statistical association: CAA mediates the relationship between Alzheimer’s pathology and microvascular injury

To rigorously delineate the mechanistic pathway linking Alzheimer’s type proteinopathy to structural vascular injury, we employed a two-tier statistical approach combining SEM and bootstrapped Causal Mediation Analysis (Table 1). The SEM framework first established the validity of the latent constructs, with Thal amyloid phase and Braak neurofibrillary tangle stage loading robustly onto the "AD Pathology" latent variable (β=0.82 and λ=0.75, respectively), and microinfarcts and white matter rarefaction converging significantly onto the "Vascular Injury" construct (λ=0.65,p<0.001). Within this structural framework, the path analysis substantiated a cascading "cross-boundary" progression: AD pathology was identified as a strong statistical antecedent associated with CAA severity (Path *a*: β=0.45,p<0.001), which in turn served as a strong correlate of concurrent vascular injury (Path *b*: β=0.12,p<0.001.). Crucially, when accounting for this indirect pathway via CAA, the direct effect of AD pathology on vascular injury (Path *c'*) was markedly attenuated (β=0.12,p<0.05), suggesting that the vascular damage traditionally attributed to AD is largely dependent on the presence of amyloid angiopathy.

**Table 1:** Integrated analysis of the "Cross-Boundary" association: structural paths and statistical mediation.

Analysis Component	Hypothesized Pathway / Relationship	Estimate	Significance	Additional Metric / Interpretation
A. Structural Paths (SEM)	AD Pathology - CAA Severity (Path a)	β = 0.45	*p* < 0.001	Strong statistical association
	CAA Severity -Vascular Injury (Path b)	β = 0.38	*p* < 0.001	Significant concurrent correlation
	AD Pathology - Vascular Injury (Direct c')	β = 0.12	*p* < 0.05	Weak direct association
	Latent Loading: Thal Phase on AD Path	*λ* = 0.82	*p* < 0.001	Construct Validity
	Latent Loading: WM Rarefaction on Vasc	*λ* = 0.65	*p* < 0.001	Construct Validity
B. Statistical Decomposition	Thal Phase - CAA - Microinfarcts			
	Indirect Effect (ACME)	0.032	*p* < 0.001	56.1 % Mediated
	Direct Effect (ADE)	0.025	*p* = 0.06	
	Total Effect	0.057	*p* < 0.001	
	APOE ϵ4 - CAA - Microinfarcts			
	Indirect Effect (ACME)	0.041	*p* < 0.001	51.9 % Mediated
	Direct Effect (ADE)	0.038	*p* = 0.04	
	Total Effect	0.079	*p* < 0.001	

Footnote: Abbreviations: AD, Alzheimer’s disease; CAA, cerebral amyloid angiopathy; SEM, structural equation modeling; WM, white matter; ACME, average causal mediation effect; ADE, average direct effect. Data in Section A are presented as standardized regression coefficients β for structural paths and factor loadings λ for latent constructs, indicating the strength of relationships adjusted for age. Data in Section B represent point estimates derived from statistical mediation analysis using 1000 bootstrap samples to determine confidence intervals and significance. The percentage mediated quantifies the proportion of the total effect of AD pathology on vascular injury that is transmitted through CAA.

To quantify the specific contribution of this intermediate pathway, statistical mediation analysis decomposed the total effects into direct and indirect components. The analysis revealed that the impact of amyloid burden on microvascular integrity is predominantly mediated by CAA. Specifically, the indirect effect via CAA accounted for 56.1 % of the association between Thal phase and cortical microinfarcts (ACME=0.032, β=0.41,p<0.001), whereas the direct effect was not statistically significant (β=0.37,p<0.001). A parallel pattern was observed for genetic risk; the APOE *ε*4 allele exerted its effect on microvascular injury primarily through the aggravation of CAA, with an estimated mediation proportion of 51.9 % (ACME=0.041, *p* < 0.001). Collectively, these findings demonstrate that CAA is not merely a comorbid feature but the obligatory conduit through which AD-associated proteinopathy and genetic risk translate into structural microvascular destruction.

### 3.7 Synergistic effects of genetic susceptibility and vascular co-pathology on cognitive dysfunction

**Fig. 4:**
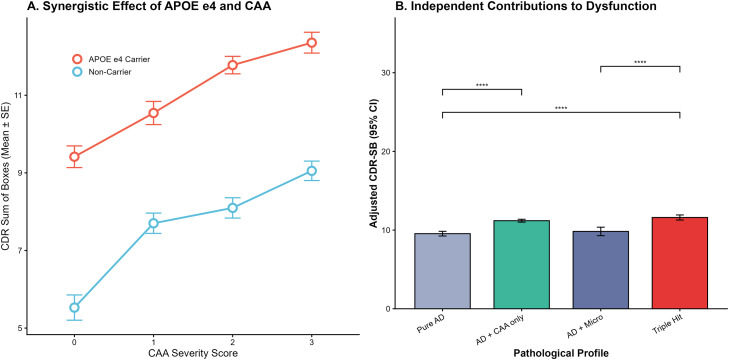
**Synergistic interaction of *APOE ε4* genotype with CAA and the cumulative impact of vascular co-pathologies on cognitive dysfunction**. **(A)** Interaction plot showing the trajectory of cognitive decline, measured by the Clinical Dementia Rating Sum of Boxes (CDR-SB), across increasing CAA severity scores. The cohort is stratified by *APOE ε4* carrier status (red) versus non-carriers (blue). Error bars represent standard errors (SE). **(B)** Bar chart displaying the adjusted mean CDR-SB scores across four mutually exclusive pathological profiles: Pure AD, AD with CAA only, AD with Microinfarcts only, and the "Triple Hit" group (concurrent AD, CAA, and Microinfarcts). Error bars represent 95 % Confidence Intervals (CI). Asterisks indicate statistical significance (** *P* < 0.01, *** *P* < 0.001, **** *P* < 0.0001).

We further investigated how genetic susceptibility modulates the clinical consequences of CAA and quantified the cumulative impact of vascular co-pathologies (Fig. 4). Panel A illustrates the interaction between APOE ε4 status and CAA severity. While cognitive decline (elevated CDR-SB) was observed with increasing CAA burden in all participants, APOE ε4 carriers exhibited a markedly more severe trajectory. Carriers started with a higher baseline impairment at CAA score 0 (Mean CDR-SB = 9.4) compared to non-carriers (Mean = 5.6) and reached the highest level of dysfunction at severe CAA stages (Mean = 12.2 vs. 9.1), indicating that the APOE ε4 allele amplifies the neurotoxic consequences of amyloid angiopathy.

Panel B delineates the independent and additive contributions of mixed pathologies across four mutually exclusive profiles. After adjusting for age, sex, and education, the "Pure AD" group exhibited a baseline adjusted mean CDR-SB score of 9.8 (95 % CI: 9.5–10.1). The presence of CAA significantly exacerbated this impairment; the "AD + CAA only" group demonstrated a mean score of 11.2 (95 % CI: 11.0–11.4), which was significantly higher than the Pure AD group (*P* < 0.0001). The "AD + Microinfarcts" group showed a mean score of 9.9 (95 % CI: 9.6–10.2). Most notably, the "Triple Hit" group—characterized by the convergence of AD, CAA, and microinfarcts—exhibited the most profound cognitive dysfunction with an adjusted mean CDR-SB of 11.5 (95 % CI: 11.2–11.8). Post-hoc comparisons confirmed that the Triple Hit group had significantly worse cognitive outcomes compared to both the Pure AD group (*P* < 0.0001) and the AD + Microinfarcts group (*P* < 0.0001), confirming that the accumulation of vascular pathology is associated with cognitive failure beyond the threshold established by AD proteinopathy alone, likely reflecting a more advanced global disease state.

## 4 Discussion

In this large-scale clinico-pathological study utilizing the NACC cohort, we provide comprehensive evidence that cerebrovascular pathology is not merely a prevalent co-morbidity in AD but acts as a critical mechanistic mediator of cognitive decline. By leveraging SEM and interaction analyses, our findings bridge the gap between the proteinopathy of AD and the structural injury of vascular disease. We demonstrate three principal insights: first, that the impact of parenchymal AD pathology on cognition is partially, yet significantly, mediated through a specific vascular cascade involving CAA and downstream cortical microinfarcts; second, that APOE ε4 status acts as a potent effect modifier, amplifying the neurotoxic consequences of CAA; and third, that the convergence of AD, CAA, and microvascular lesions constitutes a "Triple Hit" that accelerates dementia severity beyond the sum of its parts. These results challenge the traditional neurocentric view of AD, advocating instead for a model where vascular resilience is a primary determinant of clinical progression.

### 4.1 The vascular mediation pathway: from proteinopathy to structural injury

A central finding of our SEM analysis is the delineation of a sequential pathological pathway: AD neuropathology (Thal phase) -> CAA severity -> Microinfarcts -> Cognitive Dysfunction. While the association between AD and CAA is well-documented, our model quantifies the extent to which AD-related cognitive decline is driven by vascular mechanisms [16]. We observed that a substantial proportion of the effect of amyloid plaques on cognition is indirect, routed through vascular injury. This aligns with the "amyloid overflow" hypothesis, which posits that as parenchymal Aβ burden exceeds the brain's clearance capacity, Ab peptides accumulate within the perivascular drainage pathways of cortical arteries [17,18].

However, it is crucial to acknowledge the potential complexity and bidirectionality of this relationship. While our model conceptually places CAA downstream of parenchymal AD pathology, the "clearance failure" hypothesis suggests that vascular dysfunction may, in fact, serve as a primary driver of parenchymal amyloidosis [19,20]. By compromising the structural integrity of vessel walls and perivascular drainage channels, CAA may impede the efflux of interstitial fluid, effectively trapping soluble Ab within the brain parenchyma and promoting its aggregation into plaques [21,22]. Therefore, the statistical mediation observed here must be interpreted cautiously; rather than a strictly unidirectional pathway, it likely reflects a highly overlapping, vicious cycle where parenchymal and vascular amyloidosis evolve concurrently and synergistically[23].

Crucially, our model identifies microinfarcts as the terminal structural event in this vascular cascade. Unlike large territorial strokes, microinfarcts are often invisible to conventional neuroimaging, yet they represent the "tipping point" where vascular pathology translates into irreversible network disruption [24,25]. It is important to note that standard neuropathological sampling, which typically examines only a limited number of representative cortical sections, inherently underestimates the total burden of microinfarcts [26]. The fact that we observed a robust association between these sparse lesions and global cognitive status-despite this methodological "tip of the iceberg" limitation-suggests that the true impact of cumulative microvascular injury on neural network disruption is likely far greater than our estimates indicate [27]. This finding underscores that even a seemingly low burden of detected microinfarcts may signal a pervasive degradation of cortical connectivity.

### 4.2 Genetic amplification: the APOE ε4 interaction and NVU dysfunction

Our interaction analysis reveals a critical nuance in the relationship between vascular pathology and cognition: the effect of CAA is not uniform across all individuals but is significantly potentiated by the APOE genotype. We found that for a given severity of CAA [28], APOE ε4 carriers exhibited a steeper trajectory of cognitive decline compared to non-carriers. This suggests that the ε4 allele does not simply increase the risk of developing pathology, but lowers the tolerance of the brain to established vascular injury [13,29].

The biological basis for this synergistic toxicity likely extends beyond simple amyloid aggregation to the fundamental integrity of the Neurovascular Unit [30]. APOE ε4 expression has been linked to accelerated pericyte loss and the breakdown of the blood-brain barrier (BBB), potentially via the cyclophilin A-matrix metalloproteinase-9 pathway. In ε4 carriers, CAA may precipitate more severe BBB leakage, allowing neurotoxic plasma proteins (e.g., fibrinogen) to enter the parenchyma [31,32]. This extravasation can trigger a heightened perivascular inflammatory response, creating a toxic microenvironment that exacerbates neuronal injury far beyond the vessel wall. Thus, for APOE ε4 carriers, CAA represents not merely a mechanical obstruction, but a potent immunologic trigger. This "gene-environment" interaction implies that vascular risk factor management may be particularly urgent for APOE ε4 carriers to prevent the conversion of vascular pathology into clinical dementia [33].

### 4.3 The "Triple Hit" hypothesis and cumulative burden

Perhaps the most clinically relevant finding is the stepwise exacerbation of cognitive impairment observed across our pathological profiles. The "Pure AD" group, despite having high-level plaque and tangle burden, retained significantly better cognitive function than the "Triple Hit" group (AD + CAA + Microinfarcts). This observation strongly supports the concept of a "threshold model" of dementia, where the brain can often tolerate significant Alzheimer’s pathology without manifesting severe dementia, provided the vascular architecture remains intact.

The "Triple Hit" phenomenon highlights the profound cognitive impact of accumulating mixed pathologies. While this may represent a later, more advanced stage of global disease rather than a distinct synergistic pathogenic entity, it nevertheless underscores the vulnerability of the brain to concurrent neurodegenerative and vascular insults. In these patients, the brain is subjected to a multi-front assault: synaptic failure driven by oligomeric amyloid and tau, hemodynamic failure driven by CAA, and permanent structural disconnection driven by microinfarcts [34]. The significant difference in CDR-SB scores between the "AD" and "Triple Hit" groups suggests that a substantial fraction of what is clinically diagnosed as "Alzheimer’s dementia" is the functional expression of this mixed pathology [35]. Consequently, clinical trials that do not stratify for vascular co-pathology may be underpowered, as the variance in cognitive decline driven by vascular lesions could mask the potential efficacy of anti-amyloid agents [36].

### 4.4 Clinical implications in the era of anti-amyloid therapy

Our results have relevance to the current landscape of AD therapeutics, particularly the deployment of monoclonal antibodies targeting amyloid-beta. Recently approved monoclonal antibodies (e.g., lecanemab and donanemab) are associated with Amyloid-Related Imaging Abnormalities (ARIA), which are manifestations of vascular leakage triggered by the mobilization of amyloid from vessel walls, particularly in the context of CAA. Our finding that CAA and microinfarcts are strongly associated with cognitive decline generates an important hypothesis: while removing plaques is the therapeutic goal, the concurrent presence of severe CAA might theoretically pose challenges for both safety and long-term efficacy. We hypothesize that if a significant portion of cognitive decline is associated with the downstream structural effects of CAA (i.e., ischemia), removing upstream plaques in late-stage disease might yield attenuated clinical benefits if vascular integrity is already compromised. However, as the present study does not include specific clinical trial treatment or longitudinal imaging data, this remains a speculative extrapolation requiring prospective validation[37].

### 4.5 Strengths and limitations

The strength of this study lies in the use of the NACC dataset, which provides a large, multi-center sample with gold-standard neuropathological diagnoses. However, several limitations must be interpreted with caution. First and foremost, while we employed SEM to model directional pathways (AD -> CAA -> Microinfarcts), the data are inherently cross-sectional. We strictly interpret these findings as statistical associations and mediation rather than definitive proof of temporal causality or directional mechanistic pathways. As noted regarding the "clearance failure" hypothesis, biological pathways are likely bidirectional. Second, the NACC cohort represents a highly educated population, which may limit generalizability. Third, our binary categorization of *APOE* ε4 carriers included individuals with the heterozygous ε2/ε4 genotype. Because the ε2 allele is widely recognized as protective against AD, its presence might partially offset the neurotoxic or vascular risks associated with ε4. This competing effect could potentially dilute the observed effect size in the carrier group, representing a minor limitation of our classification approach. Fourth, operationalizing microinfarcts as a binary variable, while analytically necessary for structural model stability, limits our ability to capture the dose-dependent effects of high microinfarct burdens, potentially underestimating their total quantitative impact on cognitive decline.

## 5 Conclusion

In conclusion, this study elucidates the structural and genetic interdependencies that drive cognitive failure in Alzheimer’s disease. We identify a distinct pathogenic axis where AD pathology and vascular failure interact to drive cognitive decline-a pathway significantly amplified by NVU dysfunction in APOE ε4 carriers. The "Triple Hit" of concurrent AD, CAA, and microinfarcts represents a catastrophic failure of the brain's structural and functional reserve. These findings strongly argue that the future of AD treatment lies in precision medicine approaches that simultaneously target proteinopathy and fortify the neurovascular unit, particularly in genetically susceptible individuals.

## Ethics approval and consent to participate

Not applicable. All data were downloaded from the internet.

## Human ethics and consent to participate declarations

Not applicable

## Participate declaration

Not applicable

## Consent for publication

Not applicable.

## Declaration of generative AI and AI-Assisted technologies in the writing process

We confirm that we have not used any AI and AI-Assisted technologies in the writing process.

## Availability of data and materials

The data used in the present study are all publicly available at https://naccdata.org/requesting-data/nacc-data

## Funding

This work was supported by the STI2023-Major Projects (2021ZD0201801); the Key and Dominant Discipline Construction Project of the Health Commission of Guizhou Province in 2023, China; the Guizhou Provincial Science and Technology Support Program Project (General item: 2025-117); and the Guizhou Provincial Clinical Medical Research Center Construction Project - Neurological Disease Research (No: LCZX[2025]003).

## CRediT authorship contribution statement

**Mingyao You:** Writing – original draft. **Chao Tang:** Writing – original draft, Visualization. **Lianfei Liu:** Visualization. **Dengpeng Chen:** Writing – original draft, Software. **Yillin Wu:** Writing – original draft, Project administration. **Dian He:** Writing – review & editing.

## Declaration of competing interest

The authors declare that the research was conducted in the absence of any commercial or financial relationships that could be construed as a potential conflict of interest.

## Appendix Supplementary materials

Image, application 1

Image, application 2
