# Supplementary material for: The concurrent burden of Alzheimer’s pathology, cerebral amyloid angiopathy, and microinfarcts on cognitive decline
Source: J Prev Alzheimers Dis. 2026 Apr 16;13(6):100568. doi: 10.1016/j.tjpad.2026.100568 (PMC13099471; doi:10.1016/j.tjpad.2026.100568)
Supplement: Supplementary file 1 [file mmc1.pdf]

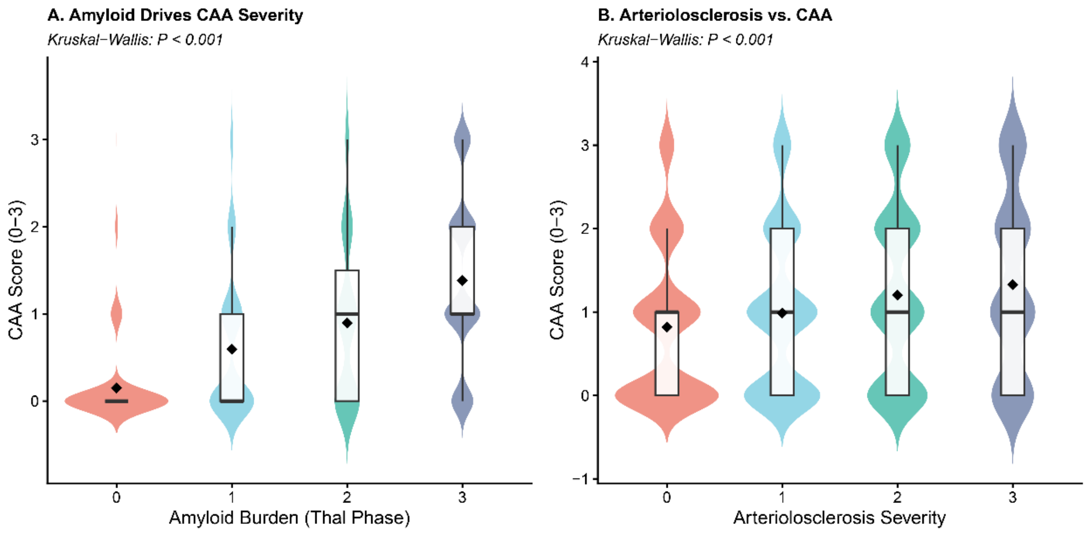

Supplementary Figure 1. Neuropathological landscape and correlational structure of Alzheimer's disease and vascular co-morbidities.

(A) Bar chart showing the prevalence of specific vascular pathologies (arteriolosclerosis, CAA, and microinfarcts) across different strata of AD neuropathologic change (ADNC). **(B)** Spearman correlation matrix quantifying the strength of associations between key neuropathological markers and global cognitive performance. The color gradient represents the correlation coefficient (Rho), with red indicating positive correlations and blue indicating negative correlations; numerical values denote the correlation coefficients.
