# Supplementary material for: The concurrent burden of Alzheimer’s pathology, cerebral amyloid angiopathy, and microinfarcts on cognitive decline
Source: J Prev Alzheimers Dis. 2026 Apr 16;13(6):100568. doi: 10.1016/j.tjpad.2026.100568 (PMC13099471; doi:10.1016/j.tjpad.2026.100568)
Supplement: Supplementary file 2 [file mmc2.docx]

**Supplementary Table 1. Demographic and Clinical Characteristics of the Study Cohort Stratified by CAA Status at Last Visit**

| Variable | Total(n=3791) | None (*n*=1357) | CAA (*n*=2434) | Statistic | p-value |
| --- | --- | --- | --- | --- | --- |
| Age, Mean±SD | 82.915±9.792 | 84.744±9.340 | 81.895±9.891 |  | <0.001 |
| Gender, *n* (%) |  |  |  |  | 0.071 |
| Female | 1937 (51.095) | 720 (53.058) | 1217 (50.000) |  |  |
| Male | 1854 (48.905) | 637 (46.942) | 1217 (50.000) |  |  |
| BMI, Mean±SD | 26.037±4.766 | 26.004±4.850 | 26.055±4.720 | *t*=-0.321^3^ | 0.748 |
| Education Years, Mean±SD | 15.673±3.036 | 15.860±2.949 | 15.569±3.080 | *t*=2.862^1^ | 0.004 |
| Clinical Diagnosis, *n* (%) |  |  |  | χ^2^=198.845^2^ | <0.001 |
| Dementia | 2583 (68.135) | 733 (54.016) | 1850 (76.007) |  |  |
| Normal cognition | 823 (21.709) | 442 (32.572) | 381 (15.653) |  |  |
| MCI | 367 (9.681) | 174 (12.822) | 193 (7.929) |  |  |
| Impaired-not-MCI | 18 (0.475) | 8 (0.590) | 10 (0.411) |  |  |
| Etiologic AD, *n* (%) |  |  |  | χ^2^=146.730^2^ | <0.001 |
| None | 823 (21.709) | 442 (32.572) | 381 (15.653) |  |  |
| Yes | 2968 (78.291) | 915 (67.428) | 2053 (84.347) |  |  |
| Hypertension, *n* (%) |  |  |  | χ^2^=8.523^2^ | 0.004 |
| None | 1170 (30.863) | 379 (27.929) | 791 (32.498) |  |  |
| Yes | 2621 (69.137) | 978 (72.071) | 1643 (67.502) |  |  |
| Diabetes, *n* (%) |  |  |  | χ^2^=2.507^2^ | 0.113 |
| None | 3250 (85.729) | 1147 (84.525) | 2103 (86.401) |  |  |
| Yes | 541 (14.271) | 210 (15.475) | 331 (13.599) |  |  |
| Heart Disease, *n* (%) |  |  |  | χ^2^=0.699^2^ | 0.403 |
| None | 3404 (89.792) | 1211 (89.241) | 2193 (90.099) |  |  |
| Yes | 387 (10.208) | 146 (10.759) | 241 (9.901) |  |  |
| Atrial Fibrillation, *n* (%) |  |  |  | χ^2^=10.931^2^ | 0.001 |
| None | 3026 (79.821) | 1044 (76.934) | 1982 (81.430) |  |  |
| Yes | 765 (20.179) | 313 (23.066) | 452 (18.570) |  |  |
| Stroke History, *n* (%) |  |  |  | χ^2^=1.118^2^ | 0.29 |
| None | 3547 (93.564) | 1262 (92.999) | 2285 (93.878) |  |  |
| Yes | 244 (6.436) | 95 (7.001) | 149 (6.122) |  |  |
| Hypercholesterolemia, *n* (%) |  |  |  | χ^2^=4.545^2^ | 0.033 |
| None | 1206 (31.812) | 461 (33.972) | 745 (30.608) |  |  |
| Yes | 2585 (68.188) | 896 (66.028) | 1689 (69.392) |  |  |
| APOE Genotype, *n* (%) |  |  |  | χ^2^=355.612^2^ | <0.001 |
| ε3,ε3 | 1759 (46.399) | 838 (61.754) | 921 (37.839) |  |  |
| ε3,ε2 | 330 (8.705) | 171 (12.601) | 159 (6.532) |  |  |
| ε3,ε4 | 1257 (33.157) | 286 (21.076) | 971 (39.893) |  |  |
| ε4,ε4 | 311 (8.204) | 25 (1.842) | 286 (11.750) |  |  |
| ε4,ε2 | 116 (3.060) | 26 (1.916) | 90 (3.698) |  |  |
| ε2,ε2 | 18 (0.475) | 11 (0.811) | 7 (0.288) |  |  |
| APOE ε4 Count, *n* (%) |  |  |  | χ^2^=350.453^2^ | <0.001 |
| No ε4 allele | 2114 (55.764) | 1022 (75.313) | 1092 (44.864) |  |  |
| 1 copy of ε4 allele | 1363 (35.954) | 309 (22.771) | 1054 (43.303) |  |  |
| 2 copy of ε4 allele | 314 (8.283) | 26 (1.916) | 288 (11.832) |  |  |
| AD, *n* (%) |  |  |  | χ^2^=203.235^2^ | <0.001 |
| None | 1384 (36.508) | 698 (51.437) | 686 (28.184) |  |  |
| Yes | 2407 (63.492) | 659 (48.563) | 1748 (71.816) |  |  |

*Footnote: Data are presented as mean ± standard deviation (SD) for continuous variables and n (%) for categorical variables. P-values were calculated using independent samples t-test for continuous variables and Pearson’s χ² test for categorical variables. CAA = Cerebral Amyloid Angiopathy; BMI = Body Mass Index; MCI = Mild Cognitive Impairment; AD = Alzheimer’s Disease; APOE = Apolipoprotein E. Clinical data were derived from the last available visit prior to death.*

**Supplementary Table 2. Neuropathological Findings and Cognitive Performance Stratified by CAA Status at Autopsy**

| Variable | Total(n=3791) | None (*n*=1357) | CAA (*n*=2434) | Statistic | p-value |
| --- | --- | --- | --- | --- | --- |
| Thal Phase, *n* (%) |  |  |  | χ^2^=763.653^2^ | <0.001 |
| A3 | 2378 (62.728) | 510 (37.583) | 1868 (76.746) |  |  |
| A2 | 494 (13.031) | 206 (15.181) | 288 (11.832) |  |  |
| A0 | 354 (9.338) | 311 (22.918) | 43 (1.767) |  |  |
| A1 | 565 (14.904) | 330 (24.318) | 235 (9.655) |  |  |
| Braak Stage, *n* (%) |  |  |  | χ^2^=665.611^2^ | <0.001 |
| B2 | 1039 (27.407) | 485 (35.741) | 554 (22.761) |  |  |
| B3 | 2025 (53.416) | 376 (27.708) | 1649 (67.749) |  |  |
| B1 | 644 (16.988) | 422 (31.098) | 222 (9.121) |  |  |
| B0 | 83 (2.189) | 74 (5.453) | 9 (0.370) |  |  |
| ADNC Score, *n* (%) |  |  |  | χ^2^=856.519^2^ | <0.001 |
| Intermediate ADNC | 972 (25.640) | 339 (24.982) | 633 (26.007) |  |  |
| High ADNC | 1769 (46.663) | 296 (21.813) | 1473 (60.518) |  |  |
| Not AD | 358 (9.443) | 312 (22.992) | 46 (1.890) |  |  |
| Low ADNC | 692 (18.254) | 410 (30.214) | 282 (11.586) |  |  |
| CERAD Score, *n* (%) |  |  |  | χ^2^=747.300^2^ | <0.001 |
| C1 | 536 (14.139) | 224 (16.507) | 312 (12.818) |  |  |
| C2 | 785 (20.707) | 252 (18.570) | 533 (21.898) |  |  |
| C3 | 1695 (44.711) | 305 (22.476) | 1390 (57.108) |  |  |
| C0 | 775 (20.443) | 576 (42.447) | 199 (8.176) |  |  |
| Diffuse Plaques, *n* (%) |  |  |  | χ^2^=700.689^2^ | <0.001 |
| Frequent diffuse plaques | 2246 (59.246) | 472 (34.783) | 1774 (72.884) |  |  |
| Moderate diffuse plaques | 630 (16.618) | 264 (19.455) | 366 (15.037) |  |  |
| Sparse diffuse plaques | 481 (12.688) | 267 (19.676) | 214 (8.792) |  |  |
| No diffuse plaques | 434 (11.448) | 354 (26.087) | 80 (3.287) |  |  |
| Microinfarcts, *n* (%) |  |  |  | χ^2^=2.115^2^ | 0.146 |
| None | 2862 (75.495) | 1006 (74.134) | 1856 (76.253) |  |  |
| Yes | 929 (24.505) | 351 (25.866) | 578 (23.747) |  |  |
| Arteriolosclerosis, *n* (%) |  |  |  | χ^2^=18.308^2^ | <0.001 |
| Severe | 549 (17.092) | 154 (14.194) | 395 (18.571) |  |  |
| Moderate | 1338 (41.656) | 432 (39.816) | 906 (42.595) |  |  |
| Mild | 1325 (41.252) | 499 (45.991) | 826 (38.834) |  |  |
| Old Hemorrhage, *n* (%) |  |  |  | χ^2^=1.759^2^ | 0.185 |
| None | 3558 (93.854) | 1283 (94.547) | 2275 (93.468) |  |  |
| Yes | 233 (6.146) | 74 (5.453) | 159 (6.532) |  |  |
| WM Rarefaction, *n* (%) |  |  |  | χ^2^=3.344^2^ | 0.188 |
| Moderate | 826 (35.420) | 266 (34.367) | 560 (35.944) |  |  |
| Mild | 1172 (50.257) | 408 (52.713) | 764 (49.037) |  |  |
| Severe | 334 (14.322) | 100 (12.920) | 234 (15.019) |  |  |
| Gross Infarcts, *n* (%) |  |  |  | χ^2^=2.543^2^ | 0.111 |
| None | 3089 (81.482) | 1124 (82.830) | 1965 (80.731) |  |  |
| Yes | 702 (18.518) | 233 (17.170) | 469 (19.269) |  |  |
| Infarcts Lacunes Sum, *n* (%) |  |  |  | χ^2^=4.239^2^ | 0.04 |
| None | 3672 (96.861) | 1325 (97.642) | 2347 (96.426) |  |  |
| Yes | 119 (3.139) | 32 (2.358) | 87 (3.574) |  |  |
| Vascular Path Sum, *n* (%) |  |  |  | χ^2^=87.200^2^ | <0.001 |
| None | 48 (1.266) | 48 (3.537) | 0 (0.000) |  |  |
| Yes | 3743 (98.734) | 1309 (96.463) | 2434 (100.000) |  |  |
| Microinfarcts Cortical, *n* (%) |  |  |  | *Z*=-1.052^4^ | 0.293 |
| 0 | 3333 (87.919) | 1202 (88.578) | 2131 (87.551) |  |  |
| 1 | 245 (6.463) | 90 (6.632) | 155 (6.368) |  |  |
| 2 | 88 (2.321) | 33 (2.432) | 55 (2.260) |  |  |
| 3 or more | 125 (3.297) | 32 (2.358) | 93 (3.821) |  |  |
| Microinfarcts WhiteMatter, *n* (%) |  |  |  | *Z*=0.709^4^ | 0.478 |
| 0 | 3582 (94.487) | 1277 (94.105) | 2305 (94.700) |  |  |
| 1 | 112 (2.954) | 50 (3.685) | 62 (2.547) |  |  |
| 2 | 40 (1.055) | 13 (0.958) | 27 (1.109) |  |  |
| 3 or more | 57 (1.504) | 17 (1.253) | 40 (1.643) |  |  |
| Microinfarcts Subcortical Gray Matter, *n* (%) |  |  |  | *Z*=3.397^4^ | 0.001 |
| 0 | 3317 (87.497) | 1155 (85.114) | 2162 (88.825) |  |  |
| 1 | 251 (6.621) | 100 (7.369) | 151 (6.204) |  |  |
| 2 | 87 (2.295) | 39 (2.874) | 48 (1.972) |  |  |
| 3 or more | 136 (3.587) | 63 (4.643) | 73 (2.999) |  |  |
| Microinfarcts Brainstem, *n* (%) |  |  |  | *Z*=-0.707^4^ | 0.479 |
| 0 | 3586 (94.592) | 1288 (94.915) | 2298 (94.412) |  |  |
| 1 | 113 (2.981) | 45 (3.316) | 68 (2.794) |  |  |
| 2 | 36 (0.950) | 8 (0.590) | 28 (1.150) |  |  |
| 3 or more | 56 (1.477) | 16 (1.179) | 40 (1.643) |  |  |
| TDP43 Stage, *n* (%) |  |  |  | χ^2^=38.838^2^ | <0.001 |
| None | 2665 (70.298) | 1038 (76.492) | 1627 (66.845) |  |  |
| Yes | 1126 (29.702) | 319 (23.508) | 807 (33.155) |  |  |
| Hippocampal Scl, *n* (%) |  |  |  | χ^2^=4.345^2^ | 0.114 |
| Severe | 430 (67.504) | 129 (62.019) | 301 (70.163) |  |  |
| Mild | 92 (14.443) | 34 (16.346) | 58 (13.520) |  |  |
| Moderate | 115 (18.053) | 45 (21.635) | 70 (16.317) |  |  |
| CDR Global, M (Q_1_, Q_3_) | 1.000 (0.500, 3.000) | 0.500 (0.000, 2.000) | 2.000 (0.500, 3.000) | *Z*=-13.631^4^ | <0.001 |
| CDR Sum Boxes, M (Q_1_, Q_3_) | 9.000 (1.000, 16.000) | 4.000 (0.000, 13.000) | 11.000 (3.000, 17.000) | *Z*=-13.985^4^ | <0.001 |
| MMSE Total, M (Q_1_, Q_3_) | 19.000 (9.000, 27.000) | 25.000 (14.000, 29.000) | 16.000 (7.000, 25.000) | *Z*=15.079^4^ | <0.001 |
| Logical Memory, M (Q_1_, Q_3_) | 1.000 (0.000, 9.000) | 5.000 (0.000, 13.000) | 0.000 (0.000, 5.000) | *Z*=16.417^4^ | <0.001 |
| Trails B Time, M (Q_1_, Q_3_) | 300.000 (134.000, 300.000) | 206.000 (102.000, 300.000) | 300.000 (175.000, 300.000) | *Z*=-13.434^4^ | <0.001 |
| Digit Span Fwd, M (Q_1_, Q_3_) | 7.000 (5.000, 8.000) | 7.000 (5.000, 9.000) | 6.000 (4.000, 8.000) | *Z*=11.604^4^ | <0.001 |
| Digit Span Bwd, M (Q_1_, Q_3_) | 4.000 (2.000, 6.000) | 5.000 (3.000, 6.000) | 4.000 (2.000, 5.000) | *Z*=12.790^4^ | <0.001 |
| Fluency Veg, M (Q_1_, Q_3_) | 5.000 (1.000, 10.000) | 7.000 (3.000, 12.000) | 4.000 (1.000, 8.000) | *Z*=13.648^4^ | <0.001 |

*Footnote: Data are presented as n (%) for categorical variables and median (interquartile range, Q1–Q3) for continuous variables with non-normal distribution. P-values were calculated using Pearson’s χ² test for categorical variables and Mann-Whitney U test (Z-statistic reported) for continuous variables. CAA = Cerebral Amyloid Angiopathy; ADNC = Alzheimer’s Disease Neuropathologic Change; CERAD = Consortium to Establish a Registry for Alzheimer's Disease; WM = White Matter; TDP-43 = TAR DNA-binding protein 43; Scl = Sclerosis; CDR = Clinical Dementia Rating; MMSE = Mini-Mental State Examination. Cognitive scores reflect performance at the last clinical visit prior to death.*
